# A formal causal interpretation of the case-crossover design

**DOI:** 10.1111/biom.13749

**Published:** 2022-10-21

**Authors:** Zach Shahn, Miguel A. Hernán, James M. Robins

**Affiliations:** 1 CUNY School of Public Health, New York, New York, USA; 2 IBM Research, Yorktown Heights, New York, USA; 3 Departments of Epidemiology and Biostatistics, Harvard T.H. Chan School of Public Health, Boston, Massachusetts, USA; 4 CAUSALab, Harvard T.H. Chan School of Public Health, Boston, Massachusetts, USA

**Keywords:** case-crossover, causal inference, counterfactual framework

## Abstract

The case-crossover design of Maclure is widely used in epidemiology and other fields to study causal effects of transient treatments on acute outcomes. However, its validity and causal interpretation have only been justified under informal conditions. Here, we place the design in a formal counterfactual framework for the first time. Doing so helps to clarify its assumptions and interpretation. In particular, when the treatment effect is nonnull, we identify a previously unnoticed bias arising from strong common causes of the outcome at different person-times. We analyze this bias and demonstrate its potential importance with simulations. We also use our derivation of the limit of the case-crossover estimator to analyze its sensitivity to treatment effect heterogeneity, a violation of one of the informal criteria for validity. The upshot of this work for practitioners is that, while the case-crossover design can be useful for testing the causal null hypothesis in the presence of baseline confounders, extra caution is warranted when using the case-crossover design for point estimation of causal effects.

## 1. | INTRODUCTION

The case-crossover design (Maclure, 1991) is used in epidemiology and other fields to study causal effects of transient treatments on acute outcomes. One of its major advantages is that it only requires information from individuals who experience the outcome of interest (the cases). Another appealing feature is that under certain circumstances (which we will discuss at length) the case-crossover estimator adjusts for unobserved time invariant confounding. In a seminal application of this design (Mittleman et al., 1993), researchers obtained data on the physical activity (a transient treatment) of individuals who experienced a myocardial infarction (MI, an acute outcome). They then defined any person-times less than 1 h after vigorous activity as “treated,” and all other person-times as “untreated.” Finally, they considered each person-time as an individual observation and computed a Mantel–Haenszel estimate of the corresponding hazard ratio (Greenland & Robins, 1985; Kleinbaum et al., 1982; Nurminen, 1981; Tarone et al., 1983). This hazard ratio estimate was interpreted as the causal effect of vigorous physical activity on MI. Some variants of the case-crossover design allow flexible control time selection strategies where control times can follow outcome occurrence (e.g., Levy et al., 2001), but in this paper we restrict attention to studies in which follow-up is terminated at the time of the first outcome occurrence as in the above MI example.

Past authors have extensively considered several threats to validity of the case-crossover design (Greenland, 1996; Janes et al., 2005; Levy et al., 2001; Maclure, 1991; Mittleman & Mostofsky, 2014; Vines & Farrington, 2001), and conditions for causal interpretation of the estimator have been informally stated in the literature. The usual criteria cited are that: (a) the outcome has acute onset; (b) the treatment effect on the outcome is transient; (c) there are no unobserved post-baseline common causes of treatment and outcome;(d)there are no time trends in treatment; and (e) the treatment effect is constant across subjects.

The Mantel–Haenszel estimator was originally applied to estimate the treatment-outcome odds ratio when subjects were classified in strata sharing values of confounders V, and observed subjects in each stratum could be conceived of as independent draws from the (hypothetically) infinite stratum population. Under the assumptions that stratum-specific odds ratios are all equal and observations are independent within each stratum, the Mantel–Haenszel estimator was later proven consistent for the constant odds ratio as the number of strata approach infinity even if only a few subjects are observed in each stratum (Breslow, 1981). Since the values of the confounders V are held constant within each stratum, the constant odds ratio can be endowed with a causal interpretation if V includes all confounders. The same goes for the rate ratio (Greenland & Robins, 1985).

Maclure’s idea was to regard person-times (rather than subjects) as the units of analysis and subjects as the strata, then apply the Mantel–Haenszel estimator. As Maclure (1991) put it: “In the case-crossover design, the population base is considered to be stratified in the extreme, so there is only one individual per stratum... Use of subjects as their own controls eliminates confounding by subject characteristics that remain constant.” Analogy to past applications of the Mantel–Haenszel estimator would seem to imply that the case-crossover design eliminates baseline confounding as a source of bias assuming a constant treatment effect across subjects (informal condition (e)) and independent identically distributed observations across time within each subject. Of course, these two assumptions are unlikely to be satisfied in most research settings: the effect of treatment is rarely the same in all subjects, and variables at different person-times are typically not independent within subjects. Informal assumptions (a)–(d) can be viewed as a more plausible alternative to independent person-times, but to determine when the case-crossover estimator is asymptotically unbiased for causal effects in the presence of unobserved confounding requires a formal analysis.

Here we place the case-crossover design in a formal counterfactual causal inference framework (Rubin, 1978; Robins, 1986). Doing so helps to clarify its assumptions and interpretation. In Section 2, we introduce notation, describe the (possibly hypothetical) cohort that gives rise to the data in a case-crossover analysis, and summarize the MI study in more detail so that it can serve as a running example. In Section 3, we define a natural estimand motivated by a hypothetical randomized trial practitioners of the case-crossover design might wish to emulate. In Section 4, we state formal assumptions (mostly analogous to informal assumptions (a)–(e)) that allow us to causally interpret the limit of the case-crossover estimator and under which the limit approximates the trial estimand from Section 3. We identify and characterize a previously unnoticed bias present when there exist strong common causes of the outcomes at different times (as would seem likely in many instances) and the treatment effect is non-null. In Section 5, we discuss this bias and illustrate it with simulations. We also use our results from Section 4 to analyze sensitivity to effect heterogeneity, that is, violations of informal assumption (e). In Section 6, we conclude. Our general message to practitioners is that, while the case-crossover can be a clever way to test the null hypothesis of no causal effect in the presence of unobserved baseline confounding, its point estimates of nonnull effects can be sensitive to violations of unrealistic assumptions.

## 2 | DATA-GENERATING PROCESS

### 2.1 | Notation

While case-crossover studies only use data from subjects who experience the outcome, we will nonetheless describe a full cohort from which these subjects are drawn in order to facilitate the definition of certain concepts and quantities of interest. Consider a cohort of individuals followed from baseline(i.e.,study entry)—defined by calendar time, age, or time of some pre-defined index event—until they develop the outcome or the administrative end of follow-up, whichever occurs first. For simplicity, we assume no individual is lost to follow-up. Subjects are indexed by i,i∈{1,…,N}. Subject i is followed for at most T+1 person-times (e.g., hours) indexed by j∈{0,…,T}. For simplicity, we take T to be the same for all subjects. Let $A_{ij}$ be a binary variable taking values 0 and 1 indicating whether subject i was treated at time j. Let $Y_{ij}$ be a binary variable taking values 0 and 1 indicating whether the outcome of interest occurred in subject i before time j+1. We assume that $Y_{ij}$ is a “time to event” outcome in the sense that if $Y_{ij}=1$ then $Y_{ij^{\prime }}=1$ for all $j^{\prime }>j$. The above implies the temporal ordering $A_{ij}$, $Y_{ij}$,$A_{i(j+1)}$. Thus the outcome has an acute onset as required by informal condition (a). We define $A_{ij}=\}{?}^{\prime }$ if the event has occurred by time j.

For a time-varying variable Z, we denote by ${\bar{Z}}_{ij}$ the history $\left(Z_{i0},\ldots ,Z_{ij}\right)$ of Z in subject i up (i.e., prior) to time j+1. We will often omit the subscript i in the subsequent notation because we assume the data from different subjects i are independent and identically distributed. Let V denote a possibly multidimensional and unobserved baseline confounding variable that we assume has some population density p(v). (For notational convenience, we shall write conditional probabilities P{⋅∣⋅,V=v} given V=v as $p_{v}\{\cdot |\cdot \}$. To avoid measure theoretic subtleties, we shall henceforth assume that when V has continuous components, conditions sufficient to pick out a particular version of P{⋅∣⋅,V=v} have been imposed as in Gill and Robins (2001).) Let ${\bar{U}}_{K}$ denote common causes of outcomes (but not treatments) at different person-times not included in V. For example, in the MI and exercise study, $U_{j}$ could denote formation of a blood clot by hour j after baseline. We assume that the N subjects are 𝑖𝑖𝑑 realizations of the random vector $\left(V,{\bar{A}}_{T},{\bar{Y}}_{T},{\bar{U}}_{T}\right)$ and that $U_{j}$ precedes $A_{j}$ and $Y_{j}$ in the temporal ordering at each j. Recall that in a case-crossover study the observed data on subject i are $\left({\bar{A}}_{iT},{\bar{Y}}_{iT}\right)$ as data on V and ${\bar{U}}_{T}$ are not available.

**FIGURE 1:**
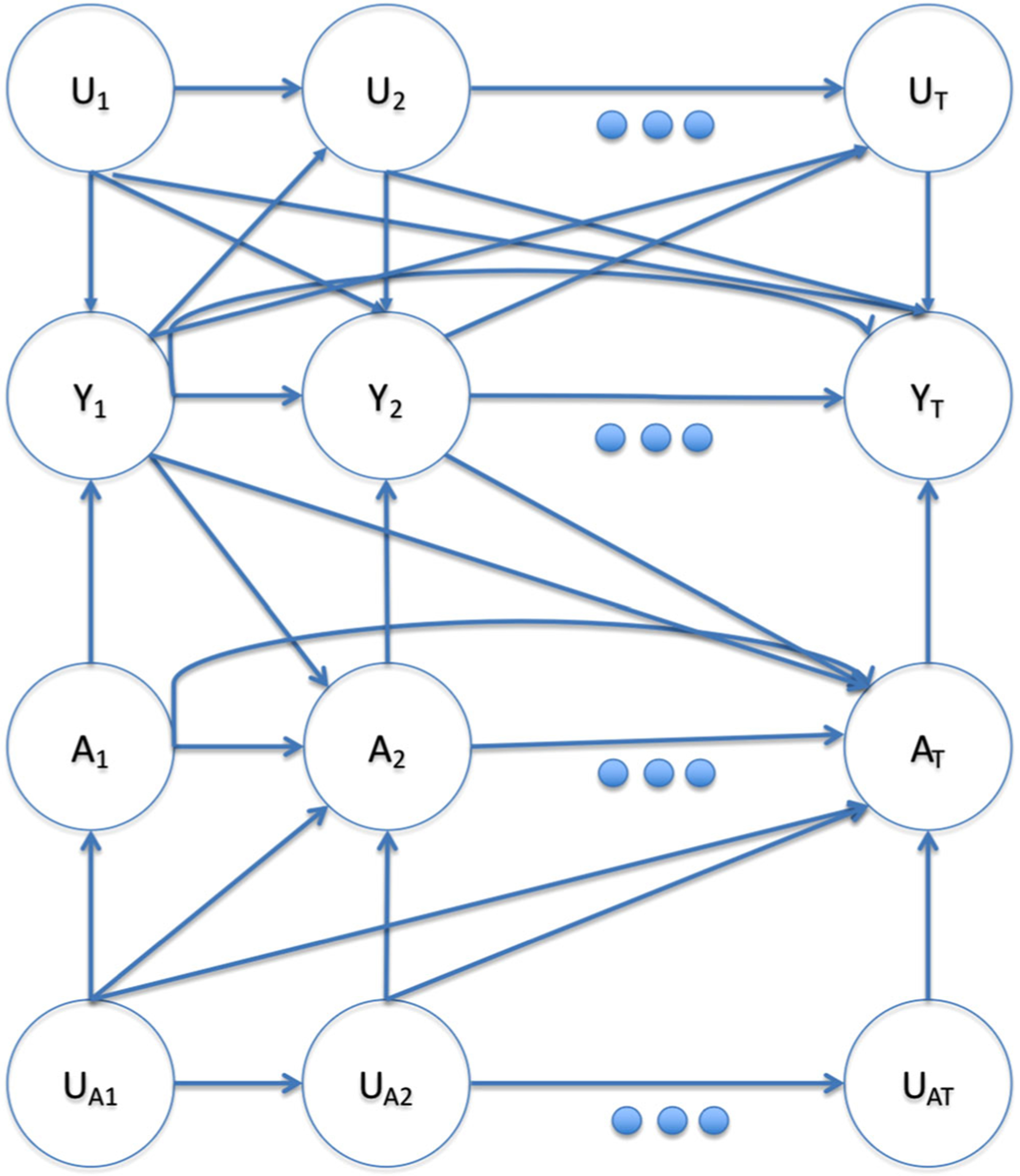
Causal DAG within levels of V. A V node with arrows pointing into every other node was omitted for visual clarity. This figure appears in color in the electronic version of this article, and any mention of color refers to that version

We assume that the causal directed acyclic graph (DAG) (Greenlandetal.,1999) in Figure 1 describes the data generating process within levels of baseline confounders V. This DAG encodes aspects of informal assumptions (b) and (c). One salient feature of the DAG is that there are no directed paths from a current treatment to an outcome at a later time that do not first pass through the outcome at the time of the current treatment or through a later treatment. This can be considered a representation of informal assumption (b) that the treatment effect is transient. The DAG also excludes any common causes of treatments and outcomes other than V not through past outcomes.(Since occurrence of the outcome at time j determines the values of all variables at all later time points, outcome variable nodes in the DAG trivially must have arrows to all temporally subsequent variables.) This represents informal assumption (c) which bars nonbaseline confounding. This DAG also has fully forward connected treatments with arbitrary common causes of treatment at different times ${\bar{U}}_{AT}$, indicating that we put no *causal* restrictions on the treatment assignment process. (We will, however, impose distributional assumptions.) We provide a fuller discussion of causal assumptions in Section 4, but we find it helpful to keep this DAG in mind.

### 2.2 | The case-crossover design

The outcome-censored case-crossover Mantel–Haenszel estimator requires data from subjects who experience the outcome on treatment status at the time of outcome occurrence and at designated “control” times preceding the outcome. It is computed as follows:

- Select a random sample of H person-times from the $H^{*}$ person times ij satisfying $Y_{ij}=1$, $Y_{i(j-1)}=0$, and j>W where W is a maximum “look back” time chosen by the investigator. We refer to these $H^{*}$ person-times when the outcome occurred for the first time and after time W as the set of “case” person-times.
- Let $i_{h}j_{h}$ denote the person-time of the hth element of the set of H sampled case person times. From the same subject $i_{h}$, select m times $\left\{j_{h}-c_{1},\ldots ,j_{h}-c_{m}\right\}$ from the W times prior to the time $j_{h}$ of subject $i_{h}^{\prime }s$ first outcome event. We call these m times the “control” person-times for subject $i_{h}$. We discuss selection of “control” times below.
- Let $A_{h}^{1}$ denote the treatment at the case time and $\left(A_{h1}^{0},\ldots ,A_{hm}^{0}\right)$ denote treatments at the m control times in subject $i_{h}$. The Mantel–Haenszel case-crossover estimator ${\hat{IRR}}_{MH}$ is

(1) $${\hat{IRR}}_{MH}=\frac{\sum_{h}\sum_{l=1}^{m}𝟙\left\{A_{h}^{1}=1,A_{hl}^{0}=0\right\}}{\sum_{h}\sum_{l=1}^{m}𝟙\left\{A_{h}^{1}=0,A_{hl}^{0}=1\right\}}.$$

Note that for subject $i_{h}$ the only data necessary to compute ${\hat{IRR}}_{MH}$ is $\left(A_{h}^{1},A_{h1}^{0},\ldots ,A_{hm}^{0}\right)$.

Intuitively, the more subjects tend to be treated at the time of the outcome but not at earlier control times as opposed to vice versa, the stronger the estimated effect of treatment. To fix ideas, we consider an example of a case-crossover study from the literature. In a simplified version of Mittleman et al. (1993) study on the impact of exercise on MI mentioned in the introduction, suppose we collect data from a random sample of patients suffering MI on a particular Sunday. We record whether each patient exercised in the hour immediately preceding their MI and whether they exercised in the same hour the day before their MI. We compute the Mantel–Haenszel case-crossover estimator (1): in the numerator is the number of subjects who exercised immediately prior to their MI but not 24 h before, and in the denominator is the number of subjects who did not exercise immediately prior to their MI but did 24 h before. Mittleman et al. estimated a ratio of 5.9 (95% CI 4.6,7.7).

Many approaches to selecting control times might be acceptable. In the MI example, the lookback window is the 24 h before the MI and there is only one control time exactly 24 h before the outcome time. So W=24, m=1, and $c_{1}=24$ in the notation above.

## 3 | A NATURAL ESTIMAND

Consider T parallel group randomized trials in which, in trial j, j=1,…,T, treatment is randomly assigned at and only at time j to all subjects who have yet to experience the outcome. Such a time j-specific trial could estimate the immediate effect of treatment at time j. To formalize, we adopt the counterfactual framework of Robins (1986). Let $Y_{ij}^{{\bar{a}}_{j}}$ be the value of the outcome at time j had, possibly contrary to fact, subject i followed treatment regime ${\bar{a}}_{j}\equiv \left(a_{1},\ldots ,a_{j}\right)$ through time j. We refer to $Y_{ij}^{{\bar{a}}_{j}}$ as a counterfactual or potential outcome. Since we will frequently consider treatment interventions at a single time point, we also introduce the notation $Y^{a_{j}}$ as shorthand for $Y^{{\bar{A}}_{j-1},a_{j}}$, that is, the counterfactual value of random variable $Y_{j}$ under observed treatment history through j−1 and treatment at time j set to $a_{j}$. The j-specific trial would yield an estimate of 𝑗-specific risk ratio or discrete hazard ratio $\beta_{j}\equiv P\left(Y_{j}^{1}=1|{\bar{Y}}_{j-1}=0\right)/P\left(Y_{j}^{0}=1|{\bar{Y}}_{j-1}=0\right)$. Until Section 5.2, we assume

(2) $$\beta \equiv \beta_{j}\;\text{constant over}j.$$

In the next section, we establish (strong) assumptions under which the case-crossover estimator approximately converges to β.

## 4 | DERIVATION OF THE COUNTERFACTUAL INTERPRETATION OF THE LIMIT OF THE CASE-CROSSOVER ESTIMATOR

### 4.1 | Assumptions

Our goal is to specify natural and near minimal assumptions that allow us to causally interpret the limit of the case- crossover estimator. Counterfactuals and the observed data are linked by the following standard assumption:

(3) $$\text{Consistency}:Y_{j}^{{\bar{A}}_{j}}=Y_{j}\;\text{for all}\;j.$$

Consistency states that the counterfactual outcomes corresponding to the observed treatment regimes are equal to the observed outcomes. Consistency is a technical assumption that has no counterpart in the informal assumptions (a)–(e) but is implicit in almost all analyses.

We assume that the causal graph in Figure 1 describes the data-generating process (Greenland et al., 1999). We will state some specific assumptions implied by the graph in counterfactual notation and also state additional assumptions. Figure 1 encodes informal assumption (c) that there are no post-baseline confounders not contained in V, that is,

$$\begin{array}{l} \text{Sequential Exchangeability}:\\ \text{For all}\;{\bar{a}}_{k},\;A_{j}⫫\left\{Y_{k}^{{\bar{a}}_{k}};k\geq j\right\}|{\bar{Y}}_{j-1}=0,{\bar{A}}_{j-1}={\bar{a}}_{j-1},V\\ =v\;\text{for all}\;j. \end{array}$$

See Appendix B for further details. Assuming consistency, (4) can be read off the Single World Intervention Graph (Richardson & Robins, 2013) for the treatment ${\bar{a}}_{T}$ associated with the causal graph of Figure 1. An example violation of (4) in the MI study would be if caffeine intake at hour j both encouraged exercise and increased MI risk at j. We might expect that confounders of this sort in the MI study (short-term encouragements to exercise that are associated with MI) are weak.

The DAG in Figure 1 also reflects informal assumption (b) that effects are transient by implying that $A_{j}$ has no direct effect on $Y_{j+1},\ldots ,Y_{T}$ not through $A_{j+1},\ldots ,A_{T}$ and $Y_{j}$ for all j. Graphically, this is the statement that the only treatment variable that is a parent of $Y_{j}$ is $A_{j}$. One might hope that the graphical definition of the transient effect assumption would be equivalent to the assumption that, conditional on V, counterfactual hazards are independent of past treatment history, that is, that $\lambda_{vj}^{{\bar{a}}_{j}}\equiv p_{v}\left(Y_{j}^{{\bar{a}}_{j}}=1|{\bar{Y}}_{j-1}^{{\bar{a}}_{j-1}}=0\right)=\lambda_{vj}^{a_{j}}\left({\bar{a}}_{j-1}\right)\equiv p_{v}\left(Y_{j}^{a_{j}}=1|{\bar{Y}}_{j-1}=0,{\bar{A}}_{j-1}={\bar{a}}_{j-1}\right)=p_{v}\left(Y_{j}=1|{\bar{Y}}_{j-1}=0,{\bar{A}}_{j-1}={\bar{a}}_{j-1},A_{j}=a_{j}\right)$ is the same for all ${\bar{a}}_{j-1}$, where the equalities all follow from (4) and (3). However, this is not generally true due to collider bias (Hernan et al., 2004) stemming from the presence of the common causes ${\bar{U}}_{j}$ of the outcomes ${\bar{Y}}_{j}$ in Figure 1 since conditional on ${\bar{Y}}_{j-1}=0$, for example, the path $A_{j-1}\to Y_{j-1}\leftarrow U_{j-1}\to Y_{j}$ is open. Because of the collider bias, a formal counterfactual definition of the transient effect assumption requires conditioning on U-histories. Specifically, let $\lambda_{vj}^{a_{j}}\left({\bar{a}}_{j-1},{\bar{u}}_{j}\right)$ denote $p_{v}\left(Y_{j}^{a_{j}}=1|{\bar{Y}}_{j-1}=0,{\bar{A}}_{j-1}={\bar{a}}_{j-1},{\bar{U}}_{j}={\bar{u}}_{j}\right)$ the conditional counterfactual hazard at time j under treatment $a_{j}$ given past treatments ${\bar{a}}_{j-1}$, common causes of outcomes ${\bar{u}}_{j}$, and baseline confounders u. As implied by Figure 1, we henceforth assume:

(5) $$\text{UV}-\text{Transient Hazards}:\lambda_{vj}^{a_{j}}\left({\bar{A}}_{j-1}={\bar{a}}_{j-1},{\bar{U}}_{j-1}={\bar{u}}_{j}\right)\;does\;not\;depend\;\;on\;{\bar{a}}_{j-1}.$$

That is, conditional on V and the history of U, the current counterfactual hazard does not depend on past treatments. This assumption is consistent with the absence of any mention of such dependence in the case-crossover literature. Biological considerations determine the plausibility of (5). In the MI study, (5) would be violated if exercise can have delayed effects of more than one hour on MI. Maclure (1991) argued delayed effects would be weak in this setting.

Under (5), we can write the counterfactual hazard $\lambda_{vj}^{a_{j}}\left({\bar{a}}_{j-1},{\bar{u}}_{j}\right)$ for any ${\bar{a}}_{j-1}$ as $\lambda_{vj}^{a_{j}}\left({\bar{u}}_{j}\right)$. Define $\lambda_{vj}^{a_{j}}=\sum_{{\bar{u}}_{j}}\lambda_{vj}^{a_{j}}\left({\bar{u}}_{j}\right)p_{v}\left({\bar{u}}_{j}|{\bar{Y}}_{j-1}=0\right)=pr\left(Y_{j}^{a_{j}}=1|{\bar{Y}}_{j-1}=0,V=v\right)$ to be the counterfactual hazard at time j given v marginal over ${\bar{U}}_{j}$. Note $\lambda_{vj}^{a_{j}}$ is the treatment arm $a_{j}$v-specific conditional risk being estimated in the RCT conducted at time j described in Section 3. Thus, $\beta_{vj}=\lambda_{vj}^{1}/\lambda_{vj}^{0}$ is the vj-specific relative risk. Define $\lambda_{j}^{a_{j}}=\int \lambda_{vj}^{a_{j}}p\left(v|{\bar{Y}}_{j-1}=0\right)dv=pr\left(Y_{j}^{a_{j}}=1|{\bar{Y}}_{j-1}=0\right)$.Then $\beta_{j}=\lambda_{j}^{1}/\lambda_{j}^{0}=\int \beta_{vj}w(v)dv$ with weight $w(v)=\lambda_{vj}^{0}p\left(v|{\bar{Y}}_{j-1}=0\right)/\lambda_{j}^{0}\cdot \beta_{j}$ is the parameter from the time j-specific RCT in (2).

We make the constant effects assumption (e) that

(6) $$\begin{array}{l} \text{Constant Causal Hazard Ratio}:\beta =\beta_{j}=\beta_{vj}\equiv \lambda_{vj}^{1}/\lambda_{vj}^{0}\\ does\;not\;depend\;on\;v\text{,} \end{array}$$

which is stronger than assumption (2). Note under (6), $\beta_{vj}=\beta_{j}$ is collapsible over v at each j so that the definition of $\beta_{j}$ does not depend on the specific variables comprising V, which we leave unspecified. Although it is well known that hazard ratios are not collapsible (Greenland, 1999) in general, in our time j-specific RCT, $\beta_{vj}$ is just the conditional risk ratio in the follow up period from time j to j+1 among those with ${\bar{Y}}_{j-1}=0$, which is collapsible under (6).

(6) is a very strong assumption unlikely to ever hold exactly. Violations can be less extreme in subpopulations, for example, subjects who exercise regularly in the MI study. We examine sensitivity to violations of (6) in Section 5.2.

We make a rare outcome assumption that holds under all levels of V, $\bar{U}$, and $\bar{A}$.

(7) $$\begin{array}{l} \text{Rare Outcome}:\prod_{j=1}^{T}\left(1-\lambda_{vj}^{a_{j}}\left({\bar{u}}_{j}\right)\right)>1-\epsilon \;\forall {\bar{u}}_{T},v,{\bar{a}}_{T}\\ and\;\epsilon \;\text{a small positive number}. \end{array}$$

A consequence of the rare outcome assumption is that (to a good approximation) collider bias induced by conditioning on ${\bar{Y}}_{j-1}$ can be neglected. Because $\left(V,\bar{U}\right)$ can be high dimensional and contain post-baseline information, it is unlikely this assumption holds in the MI study. For example, clot formation might cause a violation. But we will see that bias can be small even if this assumption fails as long as cases occurring under the violating $\left(V,\bar{U}\right)$ levels do not account for a large proportion of total cases.

Mathematically, the final assumption we will need is

(8) $$\begin{array}{l} \text{Weighted No Time Trends in Treatment}:\\ \frac{\sum_{l}\sum_{k>W}\int_{v}\lambda_{vk}^{0}p_{v}\left(A_{k}=0,A_{k-c_{l}}=1\right)\left\{\frac{p_{v}\left(A_{k}=1,A_{k-c_{l}}=0\right)}{p_{v}\left(A_{k}=0,A_{k-c_{l}}=1\right)}-1\right\}p(v)dv}{\sum_{l}\sum_{k>W}\int_{v}\lambda_{vk}^{0}p_{v}\left(A_{k}=0,A_{k-c_{l}}=1\right)p(v)}\approx 0. \end{array}$$

The left-hand side of (8) is a weighted average of time trends in treatment $\frac{p_{v}\left(A_{k}=1,A_{k-c_{l}}=0\right)}{p_{v}\left(A_{k}=0,A_{k-c_{l}}=1\right)}-1$ within levels of V, with weights equal to $\lambda_{vk}^{0}p_{v}\left(A_{k}=0,A_{k-c_{l}}=1\right)$. One way to satisfy (8) is if $\left|\frac{p_{v}\left(A_{k}=1,A_{k-c_{l}}=0\right)}{p_{v}\left(A_{k}=0,A_{k-c_{l}}=1\right)}-1\right|$ is almost always very small, but that is a very strong condition. We find it enlightening to consider a weaker trio of jointly sufficient conditions for (8).

The first of the three jointly sufficient conditions for (8) is

(9) $$\begin{array}{l} \text{No Time}-\text{Modified Confounding}:\\ \sum_{l=1}^{m}\sum_{k>W}\int_{v}\lambda_{vk}^{0}\left\{p_{v}\left(A_{k}=1,A_{k-c_{l}}=0\right)-p_{v}\left(A_{k}=0,A_{k-c_{l}}=1\right)\right\}p(v)dv\\ \approx \sum_{l=1}^{m}\sum_{k>W}\left[\int_{v}\lambda_{vk}^{0}p(v)dv\times \int_{v}\{p_{v}\left(A_{k}=1,A_{k-c_{l}}=0\right)-p_{v}\left(A_{k}=0,A_{k-c_{l}}=1\right)\}p(v)dv\right], \end{array}$$

where $k-c_{l}$ is a control time for an outcome occurring at k. A sufficient condition for (9) to hold is that, for each k and l the marginal correlation $Cou\left(p_{v}\left(A_{k}=1,A_{k-c_{l}}=0\right)-p_{v}\left(A_{k}=0,A_{k-c_{l}}=1\right),\lambda_{Vk}^{0}\right)$ is near zero between the random functions $P_{V}\left(A_{k}=1,A_{k-c_{l}}=0\right)-p_{V}\left(A_{k}=0,A_{k-c_{l}}=1\right)$ and $\lambda_{Vk}^{0}$ of V. In fact, we require only that the sum over k and l of the k-specific covariances for each control time is near zero. This condition prevents bias from so-called *time modified* baseline confounders V (Platt et al., 2009) which, by definition, are baseline confounders V that predict both (i) the hazard of an unexposed subject failing at various times k and (ii) the difference in marginal probabilities of the events $\left(A_{k}=0,A_{k-c_{l}}=1\right)$ and $\left(A_{k}=1,A_{k-c_{l}}=0\right)$. The case-crossover literature distinguishes between baseline and post-baseline confounders and says the former are allowed but not the latter. The more relevant distinction is whether a confounder has time-varying effects. To understand the issue, first consider a post-baseline confounder. We gave the example earlier of caffeine intake $\left(C_{k}\right)$ at time k impacting probability of both exercise and MI at k (more precisely, between k and k+1). $C_{k}$ is temporally a post-baseline variable as its value is realized at time 𝑘, but in the causal ordering it could be equivalent to a baseline variable if it is not influenced by past treatments. For example, coffee at time k could be equivalent to a 𝑘-hour delayed release caffeine pill at baseline. Suppose D(k)∈V is a baseline variable (like the delayed release caffeine pill) such that D(k)=1 causes $A_{k}=1$ and $Y_{k}=1$ to be more likely. D(k) would induce bias just like $C_{k}$, even though D(k)∈V is a baseline confounder that (unlike $C_{k}$) would not lead to a violation of (4). However, whenever D(k)=1, $p\left(A_{k}=1,A_{k-c_{l}}=0\right)-p\left(A_{k}=0,A_{k-c_{l}}=1\right)$ and $\lambda_{vk}^{0}$ will both be large, inducing a correlation of the sort banned by (9). Thus, (9) serves to ban time modified confounding.

The second of the jointly sufficient conditions for (8) is a sort of positivity assumption that probability of outcome occurrences and probability of discordant exposure pairs are not so negatively correlated across levels of V that they almost never co-occur.

(10) $$\text{Discordance Positivity}:\int_{v}\lambda_{vk}^{0}p_{v}\left(A_{k}=0,A_{k-c_{l}}=1\right)\;(v)dv>\delta \int_{v}\lambda_{vk}^{0}p(v)dv\;\text{for some}\;\delta >0$$

for all k,$c_{l}$ such that $k-c_{l}$ w*ould be a control time if the outcome were to occur at*k.

The last of the jointly sufficient conditions for (8) formalizes the informal assumption (d) of no time trends in treatment.

(11) $$\text{No Time Trends in Treatment}:\left[p\left(A_{k}=1,A_{k-c_{l}}=0\right)-p\left(A_{k}=0,A_{k-c_{l}}=1\right)\right]/\delta <\epsilon_{2}$$

for all $k,c_{l}$ such that $k-c_{l}$ w*ould be a control time if the outcome were to occur at* k, δ is defined in (10), and $\epsilon_{2}$ is a small positive number. (11) is essentially the marginal pairwise exchangeability assumption previously derived by Vines and Farrington (2001). Note that the assumption is marginal over V and ${\bar{U}}_{k}$ so that it is empirically checkable (apart from δ being unknown). In Web Appendix A, we discuss bias inflation from small δ due to strong negative correlation between $\lambda_{vk}^{0}$ and $p_{v}\left(A_{k}=0,A_{k-c_{l}}=1\right)$ and provide an example where the treatment time trend is very small (on an absolute scale, though not compared to δ),and yet the estimator is significantly biased. Under (11), exposure can still exhibit arbitrarily complex temporal dependence as in the DAG in Figure 1. Whether (11) holds can depend in part on how control times are chosen. In the MI study, control times 12 h prior to the outcome could be much less likely to satisfy (11) than control times 24 h prior (e.g., 2 PM the previous day would be a better control time than 2 AM the morning of an MI that occurred at 2 PM).

### 4.2 | The limit of the Mantel–Haenszel estimator

In the theorem below, we consider the probability limit $IRR_{MH}^{*}$ of ${\hat{IRR}}_{MH}$ (1) in the outcome-censored case-crossover design under an asymptotic sequence in which the full cohort, the number of cases in the cohort, and the number of sampled cases grow at similar rates, that is, N→∞,$H^{*}/N\to d_{1}>0$, and $H/H^{*}\to d_{2}>0$. We also assume subjects are *iid*.

#### Theorem 1.

Assume (3)–(8) or (3)–(7) and (9)–(11) hold for some V and $\bar{U}$. Then, under the outcome-censored case-crossover design,${\hat{IRR}}_{MH}\overset{p}{\to }IRR_{MH}^{*}\approx \beta$.

The proof is in Appendix A, along with by product so of the derivation useful for bias analysis.

## 5 | ANALYSIS OF SELECTED SOURCES OF BIAS

### 5.1 | Bias due to strong common causes of the outcome

As discussed earlier, our rare outcome assumption within levels of (possibly post-baseline and high dimensional) common causes of the outcome is novel and unreasonably strong. In this subsection, we will examine analytically and through simulations the bias that arises when it fails even under a stronger constant effect assumption that $\beta =\lambda_{vk}^{1}\left({\bar{u}}_{k}\right)/\lambda_{vk}^{0}\left({\bar{u}}_{k}\right)$ does not depend on v, k, or ${\bar{u}}_{k}$. We first consider the special case in which, at each time k, exposure is determined by an independent coin flip with success probability p. In that case, as shown in Remark 1 in Appendix A, the multiplicative bias of the case-crossover estimator is well approximated by

(12) $$\frac{\int_{v}\sum_{k>W}\sum_{{\bar{u}}_{k}}M_{v}\left({\bar{u}}_{k}\right)\left\{1-\lambda_{v,k-c}^{0}\left({\bar{u}}_{k-c}\right)\right\}p(v)dv}{\int_{v}\sum_{k>W}\sum_{{\bar{u}}_{k}}M_{v}\left({\bar{u}}_{k}\right)\left\{1-\lambda_{v,k-c}^{1}\left({\bar{u}}_{k-c}\right)\right\}p(v)dv},$$

where $M_{v}\left({\bar{u}}_{k}\right)=\lambda_{vk}^{0}\left({\bar{u}}_{k}\right)\left\{\prod_{j=1}^{k}p_{v}\left(u_{j}|{\bar{Y}}_{j-1}=0,{\bar{U}}_{j-1}={\bar{u}}_{j-1}\right)\right\}$.

Disparities between the numerator and denominator of the bias term (12) will lead to bias of the estimator. Before examining disparities related to nonnegligible V and U-specific hazards, we note that the bias contribution of a disparity at a given level of v and ${\bar{u}}_{k}$ depends on the weight $M_{v}\left({\bar{u}}_{k}\right)p(v)$, which is large when both the probability of observing $\left(v,{\bar{u}}_{k}\right)$ and the probability of an untreated event occurring at k given v and ${\bar{u}}_{k}$ are large. Thus, the larger the proportion of total cases occurring at v and ${\bar{u}}_{k}$, the more that failure of the rare outcome assumption at v and ${\bar{u}}_{k}$ biases the estimator.

The only difference between the numerator and denominator of (12) is that where $1-\lambda_{v,k-c}^{0}\left({\bar{u}}_{k-c}\right)$ appears in the numerator, $1-\lambda_{v,k-c}^{1}\left({\bar{u}}_{k-c}\right)$ appears in the denominator. The ratio of the term in the numerator to that in the demoninator is $\frac{1-\lambda_{v,k-c}^{0}\left({\bar{u}}_{k-c}\right)}{1-\beta \lambda_{v,k-c}^{0}\left({\bar{u}}_{k-c}\right)}$. When β=1, this factor is equal to 1 and there is no bias. When β≠1, the bias is away from the null since $\frac{1-\lambda_{v,k-c}^{0}\left({\bar{u}}_{k-c}\right)}{1-\beta \lambda_{v,k-c}^{0}\left({\bar{u}}_{k-c}\right)}>1$ if and only if β>1 and thus the MH estimator converges to a limit that is further from 1 than the true β and in the same direction. For the mutiplicative bias to be nonnegligible requires a violation of the rare outcome assumption in which there exist histories v, ${\bar{u}}_{k}^{*}$ for which both $M_{v}\left({\bar{u}}_{k}^{*}\right)/\sum_{{\bar{u}}_{k}}M_{v}\left({\bar{u}}_{k}\right)$ and $\beta \lambda_{v,k-c}^{0}\left({\bar{u}}_{k-c}^{*}\right)$ are nonnegligible.

We illustrate this bias with a simulation. For N=100,000 subjects, we simulated treatments and counterfactual outcomes for 24 time steps or until the first occurrence of the outcome according to the following data-generating process (DGP).

$$U_{t}∼Bernoulli(.001);\lambda_{t}^{0}\left(U_{t-1},U_{t}\right)=\min\left(1/2,.45U_{t-1}+.45U_{t}\right)\;Y_{t}^{0}∼Bernoulli\left(\lambda_{t}^{0}\left(U_{t-1},U_{t}\right)\right);\lambda_{t}^{1}\left(U_{t-1},U_{t}\right)=2\lambda_{t}^{0}\left(U_{t-1},U_{t}\right)\;Y_{t}=A_{t}Y_{t}^{1}+\left(1-A_{t}\right)Y_{t}^{0}$$

**FIGURE 2:**
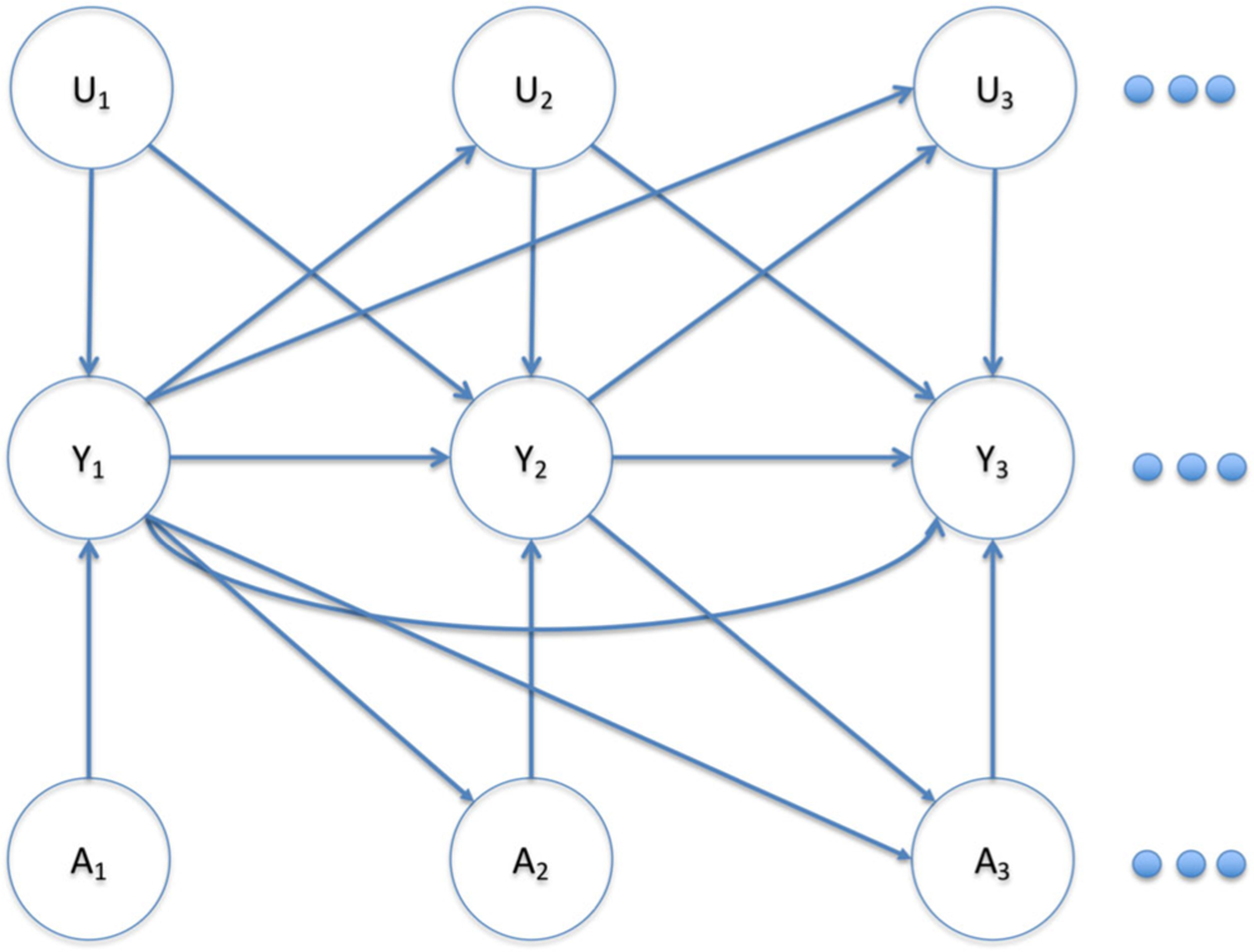
Causal DAG for simulation DGP with unobserved post-baseline common causes of outcomes at different times. This figure appears in color in the electronic version of this article, and any mention of color refers to that version

The DAG for this DGP is depicted in Figure 2. The true value of β is 2. There are no common causes of treatments and outcomes, treatments are independent identically distributed and hence exhibit no time trends, and the outcome is rare when marginalized over U. (Although the outcome is not rare when $U_{t}=1$, it is rare that $U_{t}=1$.) Yet the limit of the case-crossover estimator using the time prior to outcome occurrence as the control is approximately 2.8. The estimator fails because the outcome was common when $U_{t}$ or $U_{t-1}$ were 1 and a large proportion of total cases occurred when $U_{t}$ or $U_{t-1}$ were 1. The bias is away from the null, as predicted by our analysis above. The effect of U on the outcome needed to be strong to produce the bias in this simulation. If $\lambda_{t}^{0}\left(U_{t-1},U_{t}\right)=\min\left(1/2,.25U_{t-1}+.25U_{t}\right)$ instead of $\min\left(1/2,.45U_{t-1}+.45U_{t}\right)$, then the case-crossover estimator is about 2.3 instead of 2.8. A recently formed blood clot could roughly play the role of $\bar{U}$ in the MI example—a rare event that does not influence probability of exposure, greatly increases probability of the outcome at multiple time points after the clot forms, and without which the outcome is rare.

Now we consider bias in the more general scenario where treatments are correlated across time. In Appendix A, we expand the multiplicative bias term as

(13) $$\frac{\int_{v}\sum_{k>W}\sum_{{\bar{u}}_{k}}M_{v}\left({\bar{u}}_{k}\right)\left(1-\lambda_{v,k-c}^{0}\left({\bar{u}}_{k-c}\right)\right)\sum_{{\bar{a}}_{k/k,k-c}}G_{v}\left(1,0,{\bar{a}}_{k/k,k-c},{\bar{u}}_{k}\right)\prod_{s\neq k-c,k}\left(1-\lambda_{vs}^{a_{s}}\left({\bar{u}}_{s}\right)\right)p(v)dv}{\int_{v}\sum_{k>W}\sum_{{\bar{u}}_{k}}M_{v}\left({\bar{u}}_{k}\right)\left(1-\lambda_{v,k-c}^{1}\left({\bar{u}}_{k-c}\right)\right)\sum_{{\bar{a}}_{k/k,k-c}}G_{v}\left(0,1,{\bar{a}}_{k/k,k-c},{\bar{u}}_{k}\right)\prod_{s\neq k-c,k}\left(1-\lambda_{vs}^{a_{s}}\left({\bar{u}}_{s}\right)\right)p(v)dv},$$

where ${\bar{a}}_{k/k,k-c}$ denotes ${\bar{a}}_{k}$ excluding $a_{k}$ and $a_{k-c}$ and $G_{v}\left(a,a^{\prime },{\bar{a}}_{k/k,k-c},{\bar{u}}_{k}\right)$ (defined in Appendix A) roughly corresponds to the probability of observing treatment trajectory with $a_{k}=a,a_{k-c}=a^{\prime }$, and treatment at the other time points equal to ${\bar{a}}_{k/k,k-c}$. When treatments are correlated, $G_{v}\left(1,0,{\bar{a}}_{k/k,k-c},{\bar{u}}_{k}\right)$ in the numerator might assign high weights to different treatment sequences ${\bar{a}}_{k/k,k-c}$ than $G_{v}\left(0,1,{\bar{a}}_{k/k,k-c},{\bar{u}}_{k}\right)$ in the denominator, and under failure of the rare outcome assumption the highly weighted treatment sequences in the numerator might have significantly different survival probabilities $\left(\prod_{s\neq k-c,k}\left(1-\lambda_{vs}^{a_{s}}\left({\bar{u}}_{s}\right)\right)\right)$ for some values of v and ${\bar{u}}_{k}$ than the highly weighted treatment trajectories in the denominator. By the reasoning we applied to infer direction of bias in the case with uncorrelated exposures, strongly weighted untreated survival probabilities in the numerator combined with strongly weighted treated survival probabilities in the denominator would lead to bias away from the null, and vice versa. Depending on the treatment correlation pattern, treated or untreated survival probabilities might be more strongly weighted in the numerator or denominator. Thus, in the correlated treatment case the resulting bias can be either toward or away from the null. As in the case without correlated exposures, the magnitude of the bias contribution stemming from this dynamic for a given v and ${\bar{u}}_{k}$ depends on $M_{v}\left({\bar{u}}_{k}\right)$.

To illustrate, we modify our previous simulation example to add correlations in treatments over a time period much greater than the duration of the exposure’s transient effect. Specifically, we reduce the duration of the transient effect from 1 h to 1 s. Exposure and the unobserved common cause of the outcome are still independently assigned to 1 h intervals as in the previous simulation. This induces perfect correlation between treatments corresponding to 1 s time bins within the same hour. The untreated 1 s discrete hazards are set to preserve the hourly untreated survival probability from the previous simulation, and the multiplicative treatment effect within each one second bin is again set to 2. To formalize, we simulated data according to

$${\tilde{U}}_{k}∼Bernoulli(.001)\;\text{for}\;k\in \{1,\ldots ,24\};U_{kt}={\tilde{U}}_{k}\;\text{for}\;k\in \{1,\ldots ,24\},t\in \{1,\ldots ,3600\}$$

$${\tilde{A}}_{k}∼Bernoulli(.5)\;\text{for}\;k\in \{1,\ldots ,24\};A_{kt}={\tilde{A}}_{k}\;\text{for}\;k\in \{1,\ldots ,24\},t\in \{1,\ldots ,3600\}$$

$$\lambda_{kt}^{0}\left({\bar{U}}_{kt}\right)=0.000166\left(U_{kt}+U_{k-1t}\right);Y_{kt}^{0}∼Bernoulli\left(\lambda_{kt}^{0}\left({\bar{U}}_{kt}\right)\right);\lambda_{kt}^{1}\left({\bar{U}}_{kt}\right)=2\lambda_{kt}^{0}\left({\bar{U}}_{kt}\right)$$

$$Y_{kt}^{1}∼Bernoulli\left(\lambda_{kt}^{1}\left({\bar{U}}_{kt}\right)\right);Y_{kt}=A_{kt}Y_{kt}^{1}+\left(1-A_{kt}\right)Y_{kt}^{0}$$

where we have indexed “hours” by k and seconds within hours by t. The true value of β in this DGP is again 2, but the case-crossover estimate using the time bin exactly one hour (3600 s) prior to the case as the control (as in the previous simulation) is 1.84. So decreasing the transient exposure effect to 1 s without changing either the case-crossover estimator or the treatment duration of 1 h made the bias switch direction. The two simulations taken together illustrate that bias from strong common causes of the outcome, when present, can be both sizable and unpredictable. (See Web Appendix B for analytic confirmation of simulation results from both DGPs using (A.4), discussion of what drives the discrepancy between the two simulations, and further analysis of bias in the correlated exposure setting.)

### 5.2 | Treatment effect heterogeneity

We now examine sensitivity to violations of the constant causal hazard ratio assumption if the rare outcome assumption holds. For simplicity, we consider a scenario where there are just two types of subjects and counterfactual hazard ratios are constant across time within types. For g∈{0,1},say subjects of type g arise from the following data-generating process:

$$\begin{array}{l} A_{1},\ldots ,A_{T}\overset{id}{∼}Bernoulli\left(p_{A,g}\right);Y_{1}^{0},\ldots ,Y_{T}^{0}\overset{iid}{∼}Bernoulli\left(\lambda_{g}^{0}\right)\\ Y_{1}^{1},\ldots ,Y_{T}^{1}\overset{iid}{∼}Bernoulli\left(\lambda_{\text{g}}^{1}\right);Y_{j}=A_{j}Y_{j}^{1}+\left(1-A_{j}\right)Y_{j}^{0}, \end{array}$$

with data censored at the first occurrence of the outcome. So within each type g, the constant causal hazard ratio is $\lambda_{g}^{1}/\lambda_{g}^{0}$. Let $p_{g}$ denote the proportion of the population of type g=1 at baseline, which under the rare outcome assumption would also be approximately the proportion of type g=1 among surviving subjects at all subsequent follow-up times. According to Equation (A.6) from the proof of Theorem 1, if the rare outcome assumption holds, then the case-crossover estimator with m=1 (i.e., using just one control) will approach

(14) $$\frac{\lambda_{g=1}^{1}p_{A,g=1}\left(1-p_{A,g=1}\right)p_{g}+\lambda_{g=0}^{1}p_{A,g=0}\left(1-p_{A,g=0}\right)\left(1-p_{g}\right)}{\lambda_{g=1}^{0}p_{A,g=1}\left(1-p_{A,g=1}\right)p_{g}+\lambda_{g=0}^{0}p_{A,g=0}\left(1-p_{A,g=0}\right)\left(1-p_{g}\right)}.$$

(14) can be expressed as a weighted average of $\lambda_{g=0}^{1}/\lambda_{g=0}^{0}$ and $\lambda_{g=1}^{1}/\lambda_{g=1}^{0},\frac{\lambda_{g=0}^{1}}{\lambda_{g=0}^{0}}\frac{\delta }{\delta +\theta }+\frac{\lambda_{g=1}^{1}}{\lambda_{g=1}^{0}}\frac{\theta }{\delta +\theta }$, where $\delta =\lambda_{g=0}^{0}$ $p_{A_{g}=0}\left(1-p_{A,g=0}\right)\left(1-p_{g}\right)$ and $\theta =\lambda_{g=1}^{0}p_{A,g=1}\left(1-p_{A,g=1}\right)p_{\text{g}}$. Hence, the limit of the case-crossover estimator is bounded by the group-specific hazard ratios.

The relative risk computed from any of the RCTs described in Section 3 would approach

(15) $$\frac{\lambda_{g=1}^{1}p_{g}+\lambda_{g=0}^{1}\left(1-p_{g}\right)}{\lambda_{g=1}^{0}p_{g}+\lambda_{g=0}^{0}\left(1-p_{g}\right)}.$$

Like the case-crossover limit, the RCT estimand can be expressed as a weighted average of $\lambda_{g=0}^{1}/\lambda_{g=0}^{0}$ and $\lambda_{g=1}^{1}/\lambda_{g=1}^{0}$:

(16) $$\frac{\lambda_{g=0}^{1}}{\lambda_{g=0}^{0}}\frac{\lambda_{g=0}^{0}\left(1-p_{g}\right)}{\lambda_{g=0}^{0}\left(1-p_{g}\right)+\lambda_{g=1}^{0}p_{g}}+\frac{\lambda_{g=1}^{1}}{\lambda_{g=1}^{0}}\frac{\lambda_{g=1}^{0}p_{g}}{\lambda_{g=0}^{0}\left(1-p_{g}\right)+\lambda_{g=1}^{0}p_{g}}.$$

Without loss of generality, assume $\frac{\lambda_{g=0}^{1}}{\lambda_{g=0}^{0}}>\frac{\lambda_{g=1}^{1}}{\lambda_{g=1}^{0}}$. The ratio of the weight placed on the higher hazard ratio to the weight placed on the lower hazard ratio in the RCT estimand is

(17) $$\gamma_{RCT}\equiv \frac{\lambda_{g=0}^{0}\left(1-p_{g}\right)}{\lambda_{g=1}^{0}p_{g}}.$$

The corresponding case-crossover weight ratio is

(18) $$\begin{array}{l} \gamma_{CC}\equiv \frac{\lambda_{g=0}^{0}\left(1-p_{g}\right)p_{A,g=1}\left(1-p_{A,g=1}\right)}{\lambda_{g=1}^{0}p_{g}p_{A,g=0}\left(1-p_{A,g=0}\right)}=\gamma_{RCT}\\ \times \frac{p_{A,g=0}\left(1-p_{A,g=0}\right)}{p_{A,g=1}\left(1-p_{A,g=1}\right)}. \end{array}$$

(18) implies that bias of the case-crossover estimator due to treatment effect heterogeneity depends on the difference in treatment probability between groups with different effect sizes. If treatment probability does not vary across groups with different treatment effects, effect heterogeneity will not induce bias in the case-crossover estimator. When treatment probabilities do vary, whichever group has higher treatment variance $p_{A,g}\left(1-p_{A,g}\right)$, that is, whichever group has probability of treatment closer to 0.5, will be weighted too highly by the case-crossover estimator compared to the RCT estimand. Some intuition behind this behavior is that the closer the treatment probability within a group is to 0.5, the more subjects from that group will contribute discordant case-control pairs to the case-crossover estimator, weighting the estimator disproportionately toward the effect within that group.

For illustrative purposes, consider a numerical example where we set

$$\begin{array}{l} \lambda_{g=0}^{0}=0.001;\lambda_{g=0}^{1}=0.002;\lambda_{g=1}^{0}=0.0005;\\ \lambda_{g=1}^{1}=0.005;p_{A,g=0}=0.8;p_{A,g=1}=0.5;p_{g}=0.5. \end{array}$$

Then $\lambda_{g=1}^{1}/\lambda_{g=1}^{0}=10$, $\lambda_{g=0}^{1}/\lambda_{g=0}^{0}=2$, and the *RCT* estimand (15) is equal to 4.67. ${\hat{IRR}}_{MH}$ converges to 5.5, while the naive cohort hazard ratio estimator $\frac{P(Y=1|A=1)}{P(Y=1|A=0)}$ that does not adjust for the confounder g approaches 4.9. In this example, bias from effect heterogeneity overrides any benefits from control of unobserved confounding.

**FIGURE 3:**
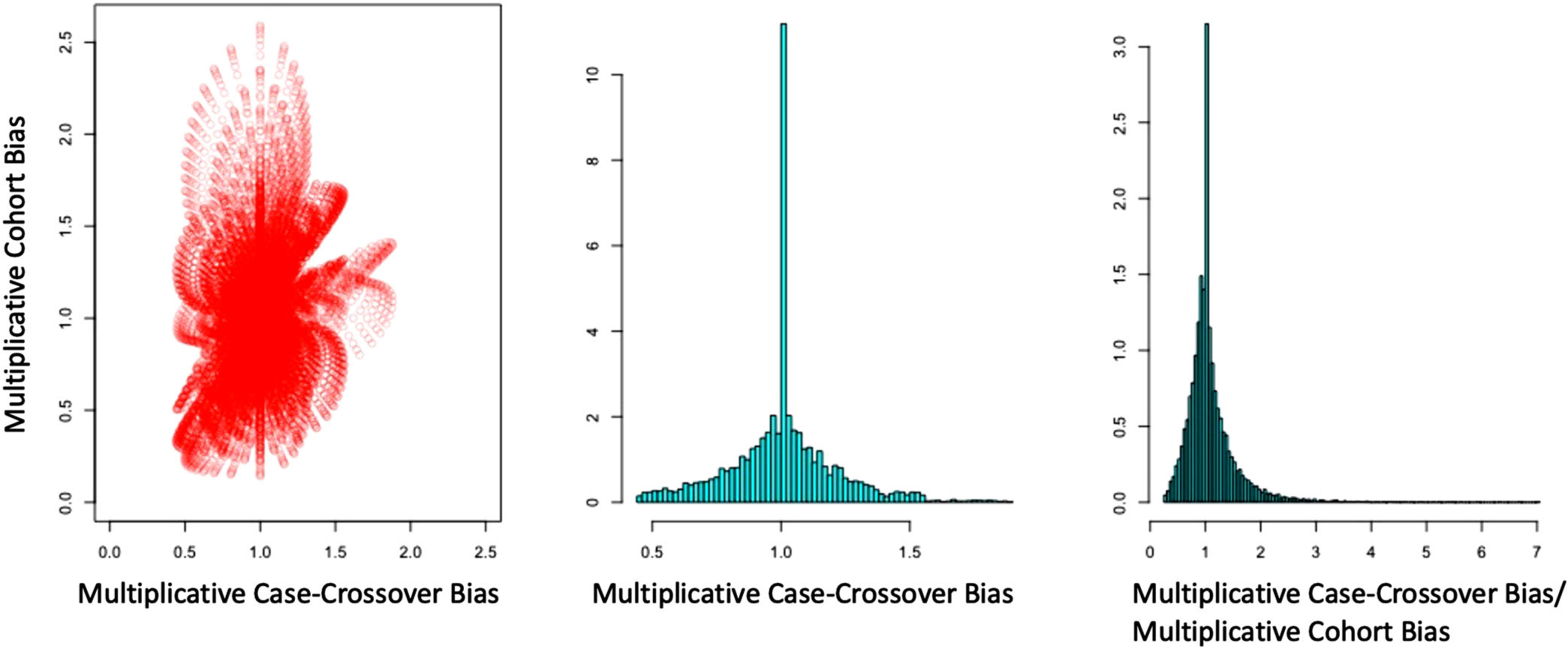
Left: Scatterplot of case-crossover versus cohort estimator multiplicative bias across a range of settings. Middle: Distribution of case-crossover estimator bias across settings. Right: Distribution of ratio of case-crossover bias to cohort bias across settings. This figure appears in color in the electronic version of this article, and any mention of color refers to that version

The specific numerical example above is a cautionary tale illustrating the potential significance of heterogeneity induced bias. But if both cohort and case-crossover analyses are feasible with available data, and unobserved baseline confounding and effect heterogeneity vary within realistic ranges, does one estimator tend to be more biased than the other? We addressed this question in the framework of our toy example by computing the limiting values of case-crossover and cohort estimators for a large grid of data-generating process parameter settings. We let $\lambda_{g=0}^{0}$ and $\lambda_{g=1}^{0}$ take values in {0.0005,0.001},$\lambda_{g=0}^{1}/\lambda_{g=0}^{0}$ take values in {1,…,5}, $\lambda_{g=1}^{1}/\lambda_{g=1}^{0}$ take values in $\left\{1\times \lambda_{g=0}^{1}/\lambda_{g=0}^{0},\ldots ,10\times \lambda_{g=0}^{1}/\lambda_{g=0}^{0}\right\}$, and $p_{A,g=0}$ and $p_{A,g=1}$ take values in {1/20,…,19/20}. Figure 3 shows that neither estimator has a general advantage over the other across parameter settings.

In the MI study, the effect of exercise appeared much greater in subjects who rarely exercised than in those who exercised regularly. Probability of treatment (i.e., exercise) clearly varied considerably between regular and rare exercise groups. Hence, we would expect an estimate of the marginal effect to be biased. The authors of the MI study reported separate effect estimates for the strata (exercise frequency prior to the study period) over which the effect was thought to vary. This is appropriate, as marginal effect estimates for the full population can be misleading.

## 6 | DISCUSSION

We have put the case-crossover estimator on more solid theoretical footing by providing a proof of its approximate convergence to a formal counterfactual causal estimand, β, under certain assumptions. This result alone may not be of much utility, but it was overdue for such a widely used method. And the derivation yielded some practical insights as by products.

First, we discovered a new source of potential bias when the treatment effect is not null–strong common causes of the outcome across time. We analyzed this bias and illustrated its potential significance and unpredictability with simulations. The effect of the common cause needs to be quite strong to induce sizable bias, but the fact that $\left(V,\bar{U}\right)$ can be high dimensional and temporally postbaseline increases the likelihood of this in a real analysis. Formation of a blood clot might induce a bias of this sort in the MI example, but it is difficult to speculate about how often meaningful bias of this type appears in practice.

Second, expression (A.6) characterizing the limit of the case-crossover estimator allowed us to quantify sensitivity to violations of the constant treatment effect assumption. We analyzed a simple scenario with two groups of subjects having potentially different baseline risks, exposure rates, and treatment effects. The limit of the case-crossover estimator was a weighted average of the group-specific hazard ratios. The bias relative to the estimand (2) that would be targeted by an RCT depends on the exposure rates in the groups. If the groups have the same exposure rate, effect heterogeneity would not induce any bias. Otherwise, whichever group had exposure rate closer to 0.5 would be overweighted. We provided a numerical example in which significant unobserved baseline confounding (which could be controlled by the case-crossover estimator) and effect heterogeneity were both present. In this example,the effect heterogeneity bias in the case-crossover estimator was greater than the confounding bias in a standard cohort hazard ratio estimator, illustrating that effect heterogeneity can sometimes override benefits from control of unobserved baseline confounding in the case-crossover estimator. More extensive numerical analyses showed that neither the cohort estimator nor the case-crossover had a general advantage across a range of settings in which the levels of unobserved confounding and effect heterogeneity varied. An analyst concerned about bias from effect heterogeneity could employ the general framework of our numerical studies to conduct a quantitative bias analysis (Lash et al., 2014).

Overall, the formal assumptions required for consistency mostly mapped onto informal assumptions (a)–(e). Unsurprisingly for a method that has been used for 30 years, our contributions do not drastically alter its recommended use. As an illustrative exercise, we assess our simplified version of Mittleman et al. (1993) study of the effect of exercise on MI assumption by assumption through the lens of our analysis in Web Appendix C.

We might summarize our general guidance to practitioners and consumers of case-crossover analyses as follows. If unobserved baseline confounding is thought to be serious and/or data collection for a cohort study is unfeasible, the case-crossover should be considered as an option. If interest lies only in testing the null hypothesis of no effect, fewer assumptions are necessary. Under the null: the transient treatment effects assumption automatically holds; common causes of the outcome do not induce bias; the rare outcome assumption is not necessary; and there is no treatment effect heterogeneity. Hence, the case-crossover design remains a clever method for causal null hypothesis testing in the presence of unmeasured baseline confounders under the exchangeability (4), no time trends in treatment (11), and no time-modified confounding (9) assumptions. If interest lies in obtaining a point estimate, results should be interpreted with considerable additional caution as effect heterogeneity, delayed treatment effects, and common causes of outcomes will all be present to some degree, and as we have shown can have a large impact on results.

There are many variants of the case-crossover design, of which we have here only analyzed arguably the simplest one. One important extension of the MH estimator adjusts for post-baseline confounders through matching. Another variant employs conditional logistic regression in place of the MH estimator. Inthiscase, Vines and Farrington(2001) showed that *joint* exchangeability is required among all control times and the case time as opposed to just pairwise exchangeability. Additionally, in situations where time trends in treatment are present, the case-time-control method (Suissa, 1995) is often utilized and requires alternative assumptions (Greenland, 1996). The case-crossover design is also frequently applied in air pollution epidemiology. In this setting, the treatment regime is shared among all subjects and later values of treatment are not influenced by past values of subjects’ outcomes, allowing more flexible control time selection strategies, including using control times following outcome occurrence (Janes et al., 2005; Levy et al., 2001; Navidi, 1998). It would be interesting to investigate these variants in a similar counterfactual framework.

## Supplementary Material

Web Appendix C (Pg 6)

## Data Availability

Data sharing is not applicable as no new data were created or analyzed in this paper.

## APPENDIX A: PROOF OF THEOREM 1

From the definition of ${\hat{IRR}}_{MH}$, it is clear that under the case-crossover design

(A.1)
$${\hat{IRR}}_{MH}\overset{p}{\to }IRR_{MH}^{*}=\frac{\sum_{l=1}^{m}Pr_{MH}\left(A^{1}=1,A_{l}^{0}=0\right)}{\sum_{l=1}^{m}Pr_{MH}\left(A^{1}=0,A_{l}^{0}=1\right)}=\frac{\sum_{l=1}^{m}Pr\left(A_{X}=1,A_{X-c_{l}}=0,X>W\right)}{\sum_{l=1}^{m}Pr\left(A_{X}=0,A_{X-c_{l}}=1,X>W\right)},$$

where X is the outcome occurrence time random variable set to ∞ if the outcome occurs after end of followup (i.e.,X>T). For simplicity, we present the proof for the case where m=1 and, if the outcome occurs at time k, there is one control time k−c (so W=c). Letting ${\bar{a}}_{k/k,k-c}$ denote ${\bar{a}}_{k}$ excluding $a_{k}$ and $a_{k-c}$, we can express (A.1) as

(A.2)
$${\text{IRR}}_{\text{MH}}^{\text{*}}=\frac{\int_{v}\sum_{k>W}\sum_{{\bar{a}}_{k/k,k-c},{\bar{u}}_{k}}p_{v}\left(Y_{k}=1,{\bar{Y}}_{k-1}=0,A_{k}=1,A_{k-c}=0,{\bar{A}}_{k/k,k-c}={\bar{a}}_{k/k,k-c},{\bar{U}}_{k}={\bar{u}}_{k}\right)p(v)dv}{\int_{v}\sum_{k>W}\sum_{{\bar{a}}_{k/k,k-c},{\bar{u}}_{k}}p_{v}\left(Y_{k}=1,{\bar{Y}}_{k-1}=0,A_{k}=0,A_{k-c}=1,{\bar{A}}_{k/k,k-c}={\bar{a}}_{k/k,k-c},{\bar{U}}_{k}={\bar{u}}_{k}\right)p(v)dv}$$

(A.3)
$$=\frac{\int_{v}\sum_{k>W}\sum_{{\bar{a}}_{k/k,k-c},{\bar{u}}_{k}}\lambda_{vk}^{1}\left({\bar{u}}_{k}\right)p_{v}\left({\bar{Y}}_{k-1}=0,A_{k}=1,A_{k-c}=0,{\bar{A}}_{k/k,k-c}={\bar{a}}_{k/k,k-c},{\bar{U}}_{k}={\bar{u}}_{k}\right)p(v)dv}{\int_{v}\sum_{k>W}\sum_{{\bar{a}}_{k/k,k-c},{\bar{u}}_{k}}\lambda_{vk}^{0}\left({\bar{u}}_{k}\right)p_{v}\left({\bar{Y}}_{k-1}=0,A_{k}=0,A_{k-c}=1,{\bar{A}}_{k/k,k-c}={\bar{a}}_{k/k,k-c},{\bar{U}}_{k}={\bar{u}}_{k}\right)p(v)dv}$$

(A.4)
$$=\frac{\sum_{k}\int_{v}\sum_{{\bar{u}}_{k}}\lambda_{vk}^{1}\left({\bar{u}}_{k}\right)p_{v}\left({\bar{U}}_{k}={\bar{u}}_{k},A_{k-c}=0,A_{k}=1,{\bar{Y}}_{k-1}=0\right)p(v)dv}{\sum_{k}\int_{v}\sum_{{\bar{u}}_{k}}\lambda_{vk}^{0}\left({\bar{u}}_{k}\right)p_{v}\left({\bar{U}}_{k}={\bar{u}}_{k},A_{k-c}=1,A_{k}=0,{\bar{Y}}_{k-1}=0\right)p(v)dv}.$$

We get (A.2) by basic probability rules; (A.3) by consistency (3), Sequential Exchangeability (4), and UV-transient hazards (5); and (A.4) by total probability. Under rare outcome assumption (7), $p_{v}\left({\bar{U}}_{k}={\bar{u}}_{k}|A_{k-c}=a,A_{k}=a^{\prime },{\bar{Y}}_{k-1}=0\right)\approx p_{v}\left({\bar{U}}_{k}={\bar{u}}_{k}|{\bar{Y}}_{k-1}=0\right)$ as collider paths| conditional on ${\bar{Y}}_{k-1}$ are effectively closed. So we can approximate (A.4)

(A.5)
$$IRR_{MH}^{*}\approx \frac{\int_{v}\sum_{k>W}\sum_{{\bar{u}}_{k}}\lambda_{vk}^{1}\left({\bar{u}}_{k}\right)p_{v}\left({\bar{U}}_{k}={\bar{u}}_{k}|{\bar{Y}}_{k-1}=0\right)p_{v}\left(A_{k}=1,A_{k-c}=0\right)p(v)dv}{\int_{v}\sum_{k>W}\sum_{{\bar{u}}_{k}}\lambda_{vk}^{0}\left({\bar{u}}_{k}\right)p_{v}\left({\bar{U}}_{k}={\bar{u}}_{k}|{\bar{Y}}_{k-1}=0\right)p_{v}\left(A_{k}=0,A_{k-c}=1\right)p(v)dv}$$

(A.6)
$$=\frac{\int_{v}\sum_{k>W}\lambda_{vk}^{1}p_{v}\left(A_{k}=1,A_{k-c}=0\right)p(v)dv}{\int_{v}\sum_{k>W}\lambda_{vk}^{0}p_{v}\left(A_{k}=0,A_{k-c}=1\right)p(v)dv}=\frac{\int_{v}\sum_{k>W}\beta_{vk}\lambda_{vk}^{0}p_{v}\left(A_{k}=1,A_{k-c}=0\right)p(v)dv}{\int_{v}\sum_{k>W}\lambda_{vk}^{0}p_{v}\left(A_{k}=0,A_{k-c}=1\right)p(v)dv}$$

(A.7)
$$=\beta \frac{\int_{v}\sum_{k>W}\lambda_{vk}^{0}p_{v}\left(A_{k}=1,A_{k-c}=0\right)p(v)dv}{\int_{v}\sum_{k>W}\lambda_{vk}^{0}p_{v}\left(A_{k}=0,A_{k-c}=1\right)p(v)dv}\equiv \beta \tau ,$$

where the first equality in (A.7) follows from the constant hazard ratio assumption (6). We can rewrite τ as

(A.8)
$$\tau =\frac{\sum_{k>W}\int_{v}\lambda_{vk}^{0}p_{v}\left(A_{k}=0,A_{k-c}=1\right)\left\{\frac{p_{v}\left(A_{k}=1,A_{k-c}=0\right)}{p_{v}\left(A_{k}=0,A_{k-c}=1\right)}-1\right\}p(v)dv}{\sum_{k>W}\int_{v}\lambda_{vk}^{0}p_{v}\left(A_{k}=0,A_{k-c}=1\right)\}p(v)}+1\approx 1\text{under (8)}.$$

Thus $IRR_{MH}^{*}\approx \beta$ under (3)–(8). Alternatively, under (9)–(11) we can write τ as

(A.9)
$$\tau =\frac{\sum_{k>W}\int_{v}\lambda_{vk}^{0}\left[p_{v}\left(A_{k}=0,A_{k-c}=1\right)+\left(p_{v}\left(A_{k}=1,A_{k-c}=0\right)-p_{v}\left(A_{k}=0,A_{k-c}=1\right)\right)\right]p(v)dv}{\sum_{k>W}\int_{v}\lambda_{vk}^{0}p_{v}\left(A_{k}=0,A_{k-c}=1\right)p(v)dv}$$

(A.10)
$$=1+\frac{\sum_{k>W}\int_{v}\lambda_{vk}^{0}\left(p_{v}\left(A_{k}=1,A_{k-c}=0\right)-p_{v}\left(A_{k}=0,A_{k-c}=1\right)\right)p(v)dv}{\sum_{k>W}\int_{v}\lambda_{vk}^{0}p_{v}\left(A_{k}=0,A_{k-c}=1\right)p(v)dv}$$

(A.11)
$$=1+\frac{\sum_{k>W}\int_{v}\lambda_{vk}^{0}p(v)dv\int_{v}\left(p_{v}\left(A_{k}=1,A_{k-c}=0\right)-p_{v}\left(A_{k}=0,A_{k-c}=1\right)\right)p(v)dv}{\sum_{k>W}\int_{v}\lambda_{vk}^{0}p_{v}\left(A_{k}=0,A_{k-c}=1\right)p(v)dv}$$

(A.12)
≈1,

where (A.9) and (A.10) are algebra, (A.11) follows from (9), and (A.12) follows from applying (10) to the denominator of the quotient in (A.11) and then (11) to the numerator, proving that $IRR_{MH}^{*}\approx \beta$ under (3)–(7) and (9)–(11).

### *Remark* 1.

In the absence of rare disease and under the stronger constant effects assumption that $\beta =\lambda_{vk}^{1}\left({\bar{u}}_{k}\right)/\lambda_{vk}^{0}\left({\bar{u}}_{k}\right)$ does not depend on v, k, or ${\bar{u}}_{k}$, it follows from (A.4) that we can expand the multiplicative bias as

(A.13)
$$\frac{\int_{v}\sum_{k>W}\sum_{{\bar{u}}_{k}}M_{v}\left({\bar{u}}_{k}\right)\left(1-\lambda_{v,k-c}^{0}\left({\bar{u}}_{k-c}\right)\right)\sum_{{\bar{a}}_{k/k,k-c}}G_{v}\left(1,0,{\bar{a}}_{k/k,k-c},{\bar{u}}_{k}\right)\prod_{s\neq k-c,k}\left(1-\lambda_{vs}^{a_{s}}\left({\bar{u}}_{s}\right)\right)p(v)dv}{\int_{v}\sum_{k>W}\sum_{{\bar{u}}_{k}}M_{v}\left({\bar{u}}_{k}\right)\left(1-\lambda_{v,k-c}^{1}\left({\bar{u}}_{k-c}\right)\right)\sum_{{\bar{a}}_{k/k,k-c}}G_{v}\left(0,1,{\bar{a}}_{k/k,k-c},{\bar{u}}_{k}\right)\prod_{s\neq k-c,k}\left(1-\lambda_{vs}^{a_{s}}\left({\bar{u}}_{s}\right)\right)p(v)dv},$$

where $M_{v}\left({\bar{u}}_{k}\right)\equiv \lambda_{vk}^{0}\left({\bar{u}}_{k}\right)\prod_{j=1}^{k}p_{v}\left(u_{j}|{\bar{Y}}_{j-1}=0,{\bar{U}}_{j-1}={\bar{u}}_{j-1}\right)$ and $G_{v}\left(a,a^{\prime },{\bar{a}}_{k/k,k-c},{\bar{u}}_{k}\right)\equiv p_{v}\left(A_{k}=a|{\bar{Y}}_{k-1}=0,A_{k-c}=a^{\prime },{\bar{A}}_{k/k,k-c}={\bar{a}}_{k/k,\;k-c},{\bar{U}}_{k}={\bar{u}}_{k}\right)\times \prod_{s=k-c+1}^{k-1}p_{v}\left(a_{s}|{\bar{Y}}_{s-1}=0,A_{k-c}=a^{\prime },{\bar{A}}_{s-1/k-c}={\bar{a}}_{s-1/k-c},{\bar{U}}_{k-c}={\bar{u}}_{k-c}\right)\times p_{v}\;\left(A_{k-c}=a^{\prime }|{\bar{Y}}_{k-c-1}=0,{\bar{A}}_{k-c-1}={\bar{a}}_{k-c-1},{\bar{U}}_{k-c-1}={\bar{u}}_{k-c-1}\right)\prod_{s=1}^{k-c-1}p_{v}\left(a_{s}|{\bar{Y}}_{s-1}=0,{\bar{A}}_{s-1}={\bar{a}}_{s-1},{\bar{U}}_{s}={\bar{u}}_{s}\right)$. If at each time s, $A_{s}\overset{\text{iid}}{∼}Bernoulli(p)$, the bias is approximately $\frac{\int_{v}\sum_{k>W}\sum_{{\bar{u}}_{k}}M_{v}\left({\bar{u}}_{k}\right)\left\{1-\lambda_{v,k-c}^{0}\left({\bar{u}}_{k-c}\right)\right\}p(v)dv}{\int_{v}\sum_{k>W}\sum_{{\bar{u}}_{k}}M_{v}\left({\bar{u}}_{k}\right)\left\{1-\lambda_{v,k-c}^{1}\left({\bar{u}}_{k-c}\right)\right\}p(v)dv}$.

## APPENDIX B: FURTHER DETAILS ON SEQUENTIAL EXCHANGEABILITY ASSUMPTION

More precisely, Equation (4) holds under the assumption (which we assume is true) that the causal DAG in Figure 1 represents an underlying *FFRCISTG* counterfactual causal model (Robins, 1986) and thus also under Pearl’s NPSEM with independent errors. See Richardson and Robins (2013) and Shpitser, Richardson, and Robins (2020).
