# Supplementary material for: A formal causal interpretation of the case-crossover design
Source: Biometrics. Author manuscript; Available in PMC 2024 May 23. (PMC11115970; doi:10.1111/biom.13749)
Supplement: Web Appendix C (Pg 6) [file NIHMS1983244-supplement-Web_Appendix_C__Pg_6_.pdf]

BIOMETRICS 64, ??–?? DOI: 10.1111/j.1541-0420.2005.00454.x
December 2008

Supporting Information for 'A Formal Counterfactual Treatment of the Case-Crossover Design' by Zach Shahn, James Robins, and Miguel Hernan

Zach Shahn[1,2,*], Miguel Hernan[3,4], and James Robins[3,4]

[1]CUNY School of Public Health, New York, NY, U.S.A

[2]IBM Research Yorktown Heights, NY, U.S.A

[3]Department of Epidemiology, Harvard University T H Chan School of Public Health, Boston, MA, U.S.A.

[4]CAUSALab, Harvard T.H. Chan School of Public Health, Boston, MA, U.S.A.

*email: Zachary.Shahn@sph.cuny.edu

This paper has been submitted for consideration for publication in *Biometrics*

Web Appendix A: Discussion of the Discordance Positivity Assumption and Sensitivity to Violations of No Time Trends In Treatment

We will assume that assumptions (3)-(7) and (9)-(10) hold and consider violations of the no time trends in treatment assumption (11) relative to the quantity δ from the discordance positivity assumption (10). Equation (29) for from the derivation of Theorem 1 gives the multiplicative bias of the case-crossover assumption under these assumptions as

$$1 + \frac{\sum_{k>W} \int_v \lambda^0_{vk} p(v)dv \int_v (p_v(A_k = 1, A_{k-c} = 0) - p_v(A_k = 0, A_{k-c} = 1))p(v)dv}{\sum_{k>W} \int_v \lambda^0_{vk} p_v(A_k = 0, A_{k-c} = 1)p(v)dv}.$$

To simplify, we will ignore the sum over k and consider a case-crossover estimator for the effect β_k at a single time k, so the multiplicative bias factor (29) becomes

$$1 + \frac{\int_v \lambda^0_{vk} p(v)dv \int_v (p_v(A_k = 1, A_{k-c} = 0) - p_v(A_k = 0, A_{k-c} = 1))p(v)dv}{\int_v \lambda^0_{vk} p_v(A_k = 0, A_{k-c} = 1)p(v)dv}. \tag{S1}$$

Looking at this formula, it is clear that bias from time trends in treatment $\int_v (p_v(A_k = 1, A_{k-c} = 0) - p_v(A_k = 0, A_{k-c} = 1))p(v)dv$ is inflated by the factor

$$\omega = \frac{\int_v \lambda^0_{vk} p(v)dv}{\int_v \lambda^0_{vk} p_v(A_k = 0, A_{k-c} = 1)p(v)dv}.$$

If λ^0_{vk} and $p_v(A_k = 0, A_{k-c} = 1)$ are strongly negatively correlated across levels of V, then ω will be large and small time trends in treatment can lead to large biases.

Consider the following simple two time step data generating process. For $j = 0, 1$,

$$V \sim Bernoulli(\theta)$$

$$A_j | Y_{j-1} = 0, V = v \sim Bernoulli(p_{vj})$$

$$\lambda^1_{vj} = \beta \lambda^0_{vj}$$

Let $\theta = 1/2$, $p_{00} = 10^{-10}$, $p_{01} = 10^{-10} + 0.01$, $p_{10} = 0.5$, $p_{11} = 0.51$, $\lambda^0_{00} = \lambda_{01} = 10^{-3}$, $\lambda^0_{10} = \lambda_{11} = 10^{-10}$, and $\beta = 1$ so that the null holds. Under this DGP, there is a small time trend in treatment as $p_{v1} - p_{v0} = .01$ for each value of v. Specifically, $\int_v (p_v(A_1 = 1, A_0 =$

$0) - p_v(A_1 = 0, A_0 = 1))p(v)dv$ is equal to

$$\begin{aligned}&(1-\theta)[(1-p_{00})(1-\lambda_{00})p_{01} - p_{00}(1-\beta\lambda_{00})(1-p_{01})]+\\ &\theta[(1-p_{10})(1-\lambda_{10})p_{11} - p_{10}(1-\beta\lambda_{10})(1-p_{11})],\end{aligned} \tag{S2}$$

which is approximately .01 under the parameter values specified above. And because the outcome is rare, (9) really does hold in that

$$\int_v \lambda^0_{v1}[p_v(A_1 = 1, A_0 = 0) - p_v(A_1 = 0, A_0 = 1)]p(v)dv \approx (1-\epsilon) \times .01 \times \int_v \lambda^0_{v1} p(v)dv$$

where ϵ is from the rare outcome assumption (7). Further, λ^0_{v1} and $p_v(A_1 = 0, A_0 = 1)$ are also strongly negatively correlated. Whenever λ^0_{v1} is not infinitesimal (though still rare), $p_v(A_1 = 0, A_0 = 1)$ is infinitesimal and vice versa. Specifically, $\int_v \lambda^0_{v1} p_v(A_1 = 0, A_0 = 1)p(v)dv$ is equal to

$$(1-\theta)\lambda^0_{01} p_{00}(1-\beta\lambda^0_{00})(1-p_{01}) + \theta\lambda^0_{11} p_{10}(1-\beta\lambda_{10})(1-p_{11}), \tag{S3}$$

which is approximately 10^{-11} under the parameter specifications above. And finally, $\int_v \lambda^0_{v1} p(v)dv$ is equal to $(1-\theta)\lambda^0_{01} + \theta\lambda^0_{11}$, which is about 0.0005. So the bias factor (S1) is approximately

$$1 + \frac{0.0005}{10^{-11}} 0.01 \approx 500000$$

with $\omega \approx 4.1 \times 10^7$. So bias from small trends in treatment can be inflated to an arbitrarily large degree by ω under special extreme circumstances.

Now, note that if $\beta \neq 0$ then there will be some small time trend in treatment even if $p_{v1} = p_{v0}$ for all v due to the dependence of the probability of suviving time 0 on A_0. If this is the only source of the time trend and counterfactual hazards are constant across time within levels of V, we do not need to worry that its impact is significantly inflated and under our simple DGP we can actually bound (S1) as follows. When $p_{v1} = p_{v0}$ and $\lambda^0_{v1} = \lambda^0_{v0}$ for all v, the time trends expression (S2) can be written as

$$(\beta - 1)[(1-\theta)p_0(1-p_0)\lambda^0_{00} + \theta p_1(1-p_1)\lambda^0_{10}].$$

And the denominator of ω (S3) can be written as

$$(1-\beta\lambda^0_{00})(1-\theta)p_0(1-p_0)\lambda^0_{01} + (1-\beta\lambda^0_{10})(1-\theta)p_1(1-p_1)\lambda^0_{11}.$$

Since $1 - \beta\lambda^0_{v0} > 1 - \epsilon$ by rare outcome, the ratio of (S2) to (S3) can be bounded by $(\beta - 1)/(1 - \epsilon)$. And of course the numerator of ω (i.e. $\int_v \lambda^0_{v1} p(v)dv$) is bounded by ϵ under the rare outcome assumption. Thus (S1) is bounded by $1 + \epsilon(\beta - 1)/(1 - \epsilon)$. And if we no longer assume that $\lambda^0_{v0} = \lambda^0_{v1}$ for all v, then (S1) is bounded by $1 + \epsilon(\beta - 1)\Delta/(1 - \epsilon)$ where $\Delta \equiv max_v \lambda^0_{v0}/\lambda^0_{v1}$.

Web Appendix B: Analysis of bias simulations from Section 5.1

Analytic confirmation of the results from the coarse independent exposure simulation

For $N = 100,000$ subjects, we simulated treatments and counterfactual outcomes until the first occurrence of the outcome according to the following data generating process (DGP):

$$U_t \sim Bernoulli(.001);\ \lambda^0_t(U_{t-1}, U_t) = min(1/2, .45U_{t-1} + .45U_t)$$

$$Y^0_t \sim Bernoulli(\lambda^0_t(U_{t-1}, U_t));\ \lambda^1_t(U_{t-1}, U_t) = 2\lambda^0_t(U_{t-1}, U_t)$$

$$Y^1_t \sim Bernoulli(\lambda^1_t(U_{t-1}, U_t));\ A_t \sim Bernoulli(.5);\ Y_t = A_t Y^1_t + (1 - A_t)Y^0_t$$

The true value of β is 2. There are no common causes of treatments and outcomes, treatments are independent identically distributed and hence exhibit no time trends, and the outcome is rare when marginalized over U. (While the outcome is not rare when $U_t = 1$, it is rare that $U_t = 1$.) Yet the limit of the case-crossover estimator using the time prior to outcome occurrence as the control is approximately 2.8.

By the proof of Theorem 1 in the Appendix (main text, not web), the bias of the case crossover estimator is approximately

$$\frac{\sum_k \int_v \sum_{\bar{u}_k} \lambda^0_{vk}(\bar{u}_k) p_v(\bar{U}_k = \bar{u}_k, A_{k-c} = 0, A_k = 1, \bar{Y}_{k-1} = 0)p(v)dv}{\sum_k \int_v \sum_{\bar{u}_k} \lambda^0_{vk}(\bar{u}_k) p_v(\bar{U}_k = \bar{u}_k, A_{k-c} = 1, A_k = 0, \bar{Y}_{k-1} = 0)p(v)dv}. \tag{S4}$$

In the DGP above, this expression approximately reduces to:

$$\frac{\sum_k \{\lambda^0_k(U_{k-1} = 1)p_U(1 - p_A)(1 - \lambda^0_{k-1}(U_{k-1} = 1))p_A + \lambda^0_k(U_k = 1)p_U(1 - p_A) \times 1 \times p_A}{\sum_k \{\lambda^0_k(U_{k-1} = 1)p_U p_A(1 - \lambda^1_{k-1}(U_{k-1} = 1))(1 - p_A) + \lambda^0_k(U_k = 1)p_U \times p_A \times 1 \times (1 - p_A)} \tag{S5}$$

where p_U and p_A denote the Bernoulli parameters of U_t and A_t, respectively, in the DGP. The only approximation in the above was to ignore the possibility that U_t is equal to 1 at more than one time t in the same subject, which leads to small approximation error since U_t is rarely 1. Plugging in the parameter values from the DGP, (S5) is equal to $1.55/1.1 \approx 2.8/2$, the bias factor obtained in the simulation.

Analytic confirmation of the results from the fine correlated exposure simulation

We modified the previous simulation example to add correlations in treatments across time induced by short time bins, and we saw that the bias flips direction. If time bins are interpreted as hours in the previous simulation, they are seconds in this one. Exposure and the unobserved common cause of the outcome are still randomly and independently assigned to one hour intervals as in the previous simulation. This has the effect of inducing (perfect) correlation between treatments in one second time bins within the same hour. The untreated one second discrete hazards are set to preserve the hourly untreated survival probability from the previous simulation, and the multiplicative treatment effect within each one second bin is again set to 2. To formalize, we simulated data according to

$$\tilde{U}_k \sim Bernoulli(.001) \text{ for } k \in \{1, \ldots, 24\}; U_{kt} = \tilde{U}_k \text{ for } k \in \{1, \ldots, 24\}, t \in \{1, \ldots, 3600\}$$

$$\tilde{A}_k \sim Bernoulli(.5) \text{ for } k \in \{1, \ldots, 24\}; A_{kt} = \tilde{A}_k \text{ for } k \in \{1, \ldots, 24\}, t \in \{1, \ldots, 3600\}$$

$$\lambda^0_{kt}(\bar{U}_{kt}) = 0.000166(U_{kt} + U_{k-1t}); Y^0_{kt} \sim Bernoulli(\lambda^0_{kt}(\bar{U}_{kt}));\ \lambda^1_{kt}(\bar{U}_{kt}) = 2\lambda^0_{kt}(\bar{U}_{kt})$$

$$Y^1_{kt} \sim Bernoulli(\lambda^1_{kt}(\bar{U}_{kt}));\ Y_{kt} = A_{kt}Y^1_{kt} + (1 - A_{kt})Y^0_{kt}$$

where we have indexed 'hours' by k and seconds within hours by t.

We can again confirm these results analytically. We use the shorthand $\lambda^a(U = 1)$ to denote the hazard at time kt if $U_{k-1t} = 1$ or if $U_{kt} = 1$, ignoring for the sake of convenient approximation the possibility that there are multiple hours with $\tilde{U}_k = 1$. The bias term for the case-crossover estimator (S1) approximately (again, under the simplifying assumption that there are not multiple hours with $\tilde{U}_k = 1$) reduces to:

$$\frac{\sum_{k=2}^{24}\sum_{t=1}^{3600}\{\lambda^0(U=1)p_U(1-p_A)(1-\lambda^0(U=1))^{3600}p_A(1-\lambda^1(U=1))^{n-1} + \lambda^0(U=1)(1-p_A)\times 1\times p_U p_A(1-\lambda^1(U=1))^{t-1}\}}{\sum_{k=1}^{24}\sum_{t=1}^{3600}\{\lambda^0(U=1)p_U p_A(1-\lambda^1(U=1))^{3600}(1-p_A)(1-\lambda^0(U=1))^{t-1} + \lambda^0(U=1)p_A\times 1\times p_U(1-p_A)(1-\lambda^0(U=1))^{t-1}}$$

$$= \frac{[\sum_{t=1}^{3600}(1-\lambda^1(U=1))^{t-1}][(1-\lambda^0(U=1))^{3600}+1]}{[\sum_{t=1}^{3600}(1-\lambda^0(U=1))^{t-1}][(1-\lambda^1(U=1))^{3600}+1]}.$$

where p_U and p_A denote the Bernoulli parameters of $\tilde{U}_k$ and $\tilde{A}_k$, respectively, in the DGP. Plugging in the parameter values from the DGP, this expression is equal to $.92 = 1.84/2$, the bias factor obtained in simulation.

Examining this bias approximation, we can see how the bias gets pushed toward the null. Selection on surviving the control hour when $\tilde{U}_{k-1} = 1$ leads to $1 - \lambda^0(U = 1)$ terms in the numerator and $1 - \lambda^1(U = 1)$ terms in the denominator. We argued in Section 5.1 that the discrepancy between these terms pushes the bias away from the null, as in the first simulation. Selection on surviving the portion of the case hour preceding the occurrence of the event leads to $1 - \lambda^1(U = 1)$ terms in the numerator and $1 - \lambda^0(U = 1)$ terms in the denominator, which by analogous reasoning pushes the bias toward the null. Selection on surviving the control hour only enters into the formula if $\tilde{U}_{k-1} = 1$, since risk is 0 whenever U is 0. Selection on surviving the case hour preceding the event, however, occurs whether $\tilde{U}_k = 1$ or $\tilde{U}_{k-1} = 1$. This explains how terms pushing the bias factor toward the null outweigh terms pushing the bias factor away from the null in this example.

We also consider analytically a slightly more general scenario (that includes our simulation) where for some set of times $\mathcal{S}_{k-c}$ exposure takes the same value as the control time (i.e. $A_s = A_{k-c}$ for $s \in \mathcal{S}_{k-c}$) and for another set of times $\mathcal{S}_k$ exposure takes the same value as the case time (i.e. $A_s = A_k$ for $s \in \mathcal{S}_k$). Such a situation would approximately arise if, e.g., time bins were taken to be very short, say 1 second, in our MI example. In almost all seconds near a given control (or case) time, the exposure level (i.e. whether the subject is exercising) will be the same, since exercise typically extends for some duration significantly longer than a second. Recall from the main text that bias with correlated exposures can be expressed as

$$\frac{\int_v \sum_{k>W} \sum_{\bar{u}_k} M_v(\bar{u}_k)(1 - \lambda^0_{v,k-c}(\bar{u}_{k-c})) \sum_{\bar{a}_{k/k,k-c}} G_v(1, 0, \bar{a}_{k/k,k-c}, \bar{u}_k) \prod_{s \neq k-c,k} (1 - \lambda^{a_s}_{vs}(\bar{u}_s))p(v)dv}{\int_v \sum_{k>W} \sum_{\bar{u}_k} M_v(\bar{u}_k)(1 - \lambda^1_{v,k-c}(\bar{u}_{k-c})) \sum_{\bar{a}_{k/k,k-c}} G_v(0, 1, \bar{a}_{k/k,k-c}, \bar{u}_k) \prod_{s \neq k-c,k} (1 - \lambda^{a_s}_{vs}(\bar{u}_s))p(v)dv} \tag{S6}$$

where $\bar{a}_{k/k,k-c}$ denotes $\bar{a}_k$ excluding a_k and a_{k-c} and $G_v(a, a', \bar{a}_{k/k,k-c}, \bar{u}_k)$ (defined in Ap-

pendix 1) roughly corresponds to the probability of observing treatment trajectory with $a_k = a$, $a_{k-c} = a'$, and treatment at the other time points equal to $\bar{a}_{k/k,k-c}$. Under the exposure correlation structure we are considering, $G_v(a, a', \bar{a}_{k/k,k-c}, \bar{u}_k)$ would equal 0 for any $\bar{a}_{k/k,k-c}$ such that $a_s \neq a$ for some $s \in \mathcal{S}_k$ or $a_s \neq a'$ for some $s \in \mathcal{S}_{k-c}$. Thus we could rewrite $\sum_{\bar{a}_{k/k,k-c}} G_v(1, 0, \bar{a}_{k/k,k-c}, \bar{u}_k) \prod_{s \neq k-c,k} (1 - \lambda_{vs}^{a_s}(\bar{u}_s))$ from the numerator of (S6) as

$$\prod_{s \in \mathcal{S}_{k-c}} (1 - \lambda_{vs}^{0}(\bar{u}_s)) \prod_{s \in \mathcal{S}_k} (1 - \lambda_{vs}^{1}(\bar{u}_s)) \sum_{\bar{a}_{k/k,k-c}} G_v(1, 0, \bar{a}_{k/k,k-c}, \bar{u}_k) \prod_{s \notin \{\mathcal{S}_{k-c}, \mathcal{S}_k, k-c, k\}} (1 - \lambda_{vs}^{a_s}(\bar{u}_s))$$

and $\sum_{\bar{a}_{k/k,k-c}} G_v(0, 1, \bar{a}_{k/k,k-c}, \bar{u}_k) \prod_{s \neq k-c,k} (1 - \lambda_{vs}^{a_s}(\bar{u}_s))$ from the denominator of (S6) as

$$\prod_{s \in \mathcal{S}_{k-c}} (1 - \lambda_{vs}^{1}(\bar{u}_s)) \prod_{s \in \mathcal{S}_k} (1 - \lambda_{vs}^{0}(\bar{u}_s)) \sum_{\bar{a}_{k/k,k-c}} G_v(0, 1, \bar{a}_{k/k,k-c}, \bar{u}_k) \prod_{s \notin \{\mathcal{S}_{k-c}, \mathcal{S}_k, k-c, k\}} (1 - \lambda_{vs}^{a_s}(\bar{u}_s)).$$

Examining the leading product terms of these expressions and applying the same logic we applied when inferring the direction of bias in the independent exposure case in Section 5.1, we see that there is no bias if $\beta = 1$ but if $\beta \neq 1$ then the bias might be either toward or away from the null depending on $\mathcal{S}_{k-c}$, $\mathcal{S}_k$, and values of $\lambda_{vs}^{1}(\bar{u}_s)$ and β.

Web Appendix C: Possible Violations of Assumptions in the Exercise and MI Study

It might be illustrative to assess our simplified version of Mittelman et al.'s (1993) seminal study on the impact of exercise on MI through the lens of our analysis. Recall that in the simplified version data collection occurs over the course of a single Sunday, so we take the baseline for the underlying cohort population of interest to be midnight of the preceding Saturday morning. It is worth noting that this study highlights the benefit of the case-only nature of the design in that it would be very difficult to collect the data required to perform a cohort study targeting an equivalent question.

No post-baseline confounding (Sequential Exchangeability, assumption (4)). We discussed the example of drinking coffee as a possible violation of this assumption. Caffeine might

increase energy and encourage exercise and also independently increase the risk of MI. We might reasonably hope that confounders of this sort (short term encouragements to exercise that are also associated with MI) are weak.

No direct effect of treatment on later outcomes (UV-Transient Hazards, assumption (5)). It is possible that exercise has a cumulative effect on the outcome. Two consecutive hours of exercise might cause an MI in some subjects for whom just one hour would not. Perhaps extended vigorous exercise is rare enough that the cumulative effect of exercise does not seriously impact results or their interpretation. Delayed effects of exercise are not thought to be significant.

No time modified confounding (assumption (9)). It might be that within levels of certain baseline confounders, exercise is more probable on Saturday (or Sunday) afternoon and MIs are more (or less) probable than average, even if marginal probability of exercise in the full cohort is equal on the two days. If the control hour is taken to be 24 hours before the MI, this scenario would induce bias.

No time trends in treatment (assumption (11)). Marginal probability of recent exercise varies greatly by time of day. If the control time is chosen appropriately (e.g. exactly 24 hours before the MI), then approximate pairwise exchangeability may hold. But perhaps there are reasons why exercise is generally more or less common on Sunday than Saturday (e.g. church or televised football games). This would induce time trend bias. It is difficult to imagine a mechanism for significant inflation of time trend bias discussed in Web Appendix A in this example.

Under the above assumptions (in addition to Consistency), the case-crossover could reasonably be applied to test the causal null hypothesis. To interpret the case-crossover point estimate, additional considerations are required.

Rare outcome (assumption (7)). The outcome must be rare within all levels of the baseline

confounders, exposure, and common causes of the outcome. MIs are certainly rare marginally at the level of a day, and probably also rare across levels of baseline confounders and exposures. However, we mentioned that perhaps causes of the outcome such as presence of a clot could make the outcome common, particularly under exposure. This would induce bias of the sort seen in the simulation in Section 5.1.

Constant causal hazard ratio (assumption (6)). It is highly unlikely that the multiplicative effect of exercise across hour and covariate levels is constant. While true under the null, this is a very strong assumption if the null does not hold. We saw in Section 5.2 that it is difficult to interpret the point estimate if the effect is heterogeneous.

Received October 2007. *Revised February* 2008. *Accepted March* 2008.
